# Population-Specific Carrier Frequencies in an Underrepresented Genetically Heterogeneous Population: Implications for Expanded Carrier Screening

**DOI:** 10.3390/genes17091118

**Published:** 2026-09-15

**Authors:** Predrag Noveski, Natalija Jovanovska, Ivana Maleva Kostovska, Gjorgji Bozhinovski, Sanja Kiprijanovska, Marija Terzikj, Marija Vujovikj, Emilija Shukarova Stefanovska, Aleksandar Dimovski, Dijana Plaseska-Karanfilska

**Affiliations:** Research Center for Genetic Engineering and Biotechnology “Georgi D. Efremov’’, Macedonian Academy of Sciences and Arts, 1000 Skopje, North Macedonia; pnoveski@manu.edu.mk (P.N.); natalija@manu.edu.mk (N.J.); maleva_i@manu.edu.mk (I.M.K.); gorgi.b@manu.edu.mk (G.B.); skiprijanovska@manu.edu.mk (S.K.); terzikj.marija@manu.edu.mk (M.T.); m.vujovic@manu.edu.mk (M.V.); emilija@manu.edu.mk (E.S.S.); a.dimovski@manu.edu.mk (A.D.)

**Keywords:** expanded carrier screening, at-risk couple, carrier frequency, North Macedonia population, underrepresented populations, pathogenic variants, recessive disorders

## Abstract

**Background/Objectives**: Expanded carrier screening (ECS) panels often rely on pan-ethnic databases, such as the ACMG Tier 3 panel, but their ability to capture population-specific reproductive risk is uncertain. We characterized pathogenic/likely pathogenic (P/LP) variants and at-risk-couple (ARC) probabilities in an underrepresented population from North Macedonia to inform ECS gene prioritization. **Methods**: NGS data from 1438 subjects of Macedonian, Albanian, and other local ancestry, referred for rare-disease diagnostics, were analyzed across 1795 candidate autosomal recessive/X-linked genes. P/LP variants were compared with gnomAD exomes v2.1.1 non-Finnish European frequencies (Fisher’s exact test). Gene-specific ARC probabilities ranked genes and generated cumulative ARC curves per subgroup. **Results**: We identified 2007 P/LP variants across 1096 genes. One hundred variants in 89 genes reached an allele frequency ≥ 1/200 in the Macedonian or Albanian subgroups; over one-third were absent from candidate ECS panels, mostly linked to severe or variable phenotypes. ARC saturated rapidly: genes with carrier counts ≥ 1/100 (ACMG Tier 2) accounted for over 85% of total ARC, and with ≥1/200 (ACMG Tier 3) for more than 93%. Total ARC among autosomal recessive panel genes (X-linked genes analyzed separately) was 3.42%, rising to 5.02% in the Albanian subgroup. Excluding top hypomorphic variants reduced ARC by 34–37%, highlighting variant-specific penetrance. The highest-ARC genes diverged between subgroups, with limited overlap with pan-ethnic panels. **Conclusions**: Population-specific carrier frequencies influence gene prioritization and estimated reproductive-risk probabilities for ECS. Population-tailored ECS panels that incorporate local carrier-frequency data, including embryonic/fetal-lethal genes, and account for hypomorphic alleles to avoid overestimating reproductive risk probabilities could improve the identification of couples at increased reproductive risk, particularly in underrepresented populations.

## 1. Introduction

Genetic carrier screening is an established component of reproductive medicine, enabling the identification of individuals and couples who carry pathogenic variants associated with autosomal recessive or X-linked disorders before or during pregnancy [1,2]. Traditionally, carrier screening was limited to a small number of relatively common disorders in populations with well-recognized founder mutations [3]. Advances in next-generation sequencing (NGS) have transformed this approach, enabling expanded carrier screening (ECS), in which hundreds of genes can be analyzed simultaneously irrespective of ancestry [4]. By identifying couples at increased reproductive risk, ECS supports informed reproductive decision-making and facilitates access to prenatal diagnosis, preimplantation genetic testing for monogenic disorders (PGT-M), and other reproductive options [2,4].

Developing an effective ECS panel requires balancing condition prevalence, clinical severity, and variant penetrance [5]. Current professional recommendations, including the ACMG Tier 3 panel of 113 genes, are largely based on pan-ethnic carrier frequencies derived from large genomic reference databases such as gnomAD, together with considerations of disease severity and clinical actionability [6,7].

Although these recommendations provide an important framework for clinical practice, they may not accurately reflect reproductive risk in populations with distinct demographic histories, founder effects, or population-specific pathogenic variants. Increasing evidence demonstrates substantial differences in carrier frequencies among populations, suggesting that a universal panel may overlook disorders that are relatively common locally while including genes that contribute little to reproductive risk in a particular population [8,9]. These observations have shifted attention from defining a universally applicable ECS panel toward developing evidence-based strategies for population-tailored panel design.

Population-specific genomic resources provide an effective means of addressing this challenge. The Balkan Peninsula is characterized by considerable genetic diversity resulting from a complex demographic history involving repeated migration, population isolation, and founder effects [10], yet populations from this region remain substantially underrepresented in major genomic reference databases such as gnomAD. Consequently, carrier frequencies derived from broader European populations may not accurately represent reproductive risk within individual Balkan populations. Large NGS datasets generated through routine rare-disease diagnostics offer a valuable opportunity to establish local carrier frequencies. When pathogenic alleles responsible for a participant’s primary diagnosis are excluded, these datasets provide robust estimates of population carrier frequencies and have successfully been used to develop population-specific genomic resources and screening strategies [11,12].

Optimizing ECS also requires careful consideration of which disorders should be represented. Beyond the conditions included in established recommendations such as the ACMG Tier 3 panel [6], several expert-curated initiatives have expanded gene selection to encompass severe childhood disorders or have defined systematic gene sets associated with prenatal lethality, providing a framework for identifying such variants [13,14,15,16,17,18].

In this study, we analyzed NGS data from 1438 individuals representing the major ethnic groups of North Macedonia to characterize the population-specific landscape of pathogenic and likely pathogenic variants. We compared local carrier frequencies with those reported in gnomAD, estimated gene-specific at-risk-couple (ARC) probabilities, and evaluated how population-specific gene prioritization differs from standardized pan-ethnic screening panels. Our objective was not only to characterize carrier frequencies in a genetically heterogeneous Balkan population, but also to establish a framework for evidence-based, population-tailored ECS panel design, thereby providing a general framework for integrating population genomics into the rational design of expanded carrier screening panels.

## 2. Materials and Methods

### 2.1. Study Population and NGS Enrichment

An initial cohort of 1532 patients referred for rare-disease genetic testing was analyzed by short-read NGS across two enrichment strategies. After excluding 94 subjects as relatives (in trios, only parents were retained; among siblings, only one was retained), the final cohort comprised 1438 subjects (Table 1).

The ethnic and geographic distribution of participants reflected the national population structure (Table 2). Ethnic group was assigned based on self-reported ethnicity recorded at the time of diagnostic referral. Self-reported ethnicity was treated as a social/population descriptor and was not considered equivalent to genetic ancestry, as the two may show substantial discordance [19,20,21]. Genetic ancestry inference was not performed.

The study was conducted in accordance with the Declaration of Helsinki; informed consent was obtained from all participants for storage of biological material and use of anonymized sequencing data.

### 2.2. Sequence Alignment, Variant Calling, and Annotation

All FASTQ files from the 1438 subjects were processed identically against the hg38 “no_alt” reference genome using an in-house pipeline.

Reads were aligned with BWA v0.7.17 [22], and variants were called with GATK v4.1.3.0 [23], restricted to target regions using enrichment-specific BED files (clinical exome and WES) with 25 bp padding on each side of targeted exons. Per-sample VCFs were merged into a single integrated VCF with bcftools v1.9. Next, variants were left-aligned and multiallelic sites were split into individual records using *bcftools norm*, followed by functional annotation with Ensembl VEP v115 [24]. Downstream analyses were performed in R v4.2.1.

### 2.3. Candidate Gene List Assembly

In addition to the ACMG pan-ethnic screening list, we incorporated genes from recently published curated sets associated with childhood-onset, life-limiting or disabling recessive disorders, and with conditions causing prenatal or embryonic lethality. Gene lists were drawn both directly from the literature and from PanelApp panels [25], which are derived from published evidence and undergo ongoing curation (Table 3). Only autosomal recessive (AR) or X-linked recessive (XLR) genes were retained from these sources, yielding one merged, non-redundant list of 1795 genes (Supplementary File S1). Gene symbols were harmonized to approved HGNC nomenclature.

### 2.4. Variant Annotation and Filtering Strategy

Variants were filtered using: (1) ClinVar classifications; (2) gnomAD exomes v2.1.1, gnomAD genomes v3.0, and local population frequency data; and (3) variant quality metrics. Annotation was performed either with VEP using custom reference files or with custom R scripts applied to the raw variant calls.

We have downloaded ClinVar.vcf and submission_summary.txt files v.20251228. The Clinvar VCF file was used for variant annotation with VEP, while the submission_summary.txt was used in R to annotate the number of times the variant was annotated as pathogenic/likely pathogenic (P/LP), variant of uncertain significance (VUS), and benign/likely benign (B/LB), and to record the first and last classification submission dates. All variants with ClinVar final P/LP classification were retained. For variants classified as “conflicting classifications of pathogenicity” with at least one P/LP submission, two filtering approaches were applied: first, variants where non-P/LP submissions outnumbered P/LP submissions were removed unless an independent automated ACMG classification with GeneBe [26] also predicted P/LP, in which case they were retained; second, variants with both P/LP and B/LB submissions were retained only if P/LP submissions outnumbered other classifications at least 2:1 and no more than one benign submission was present.

This classification workflow relied on ClinVar-submitted ACMG/AMP pathogenicity evidence and did not incorporate reduced penetrance or variable expressivity as classification criteria; a variant’s P/LP status therefore reflects its evidence for causing disease when penetrant, not the probability that a carrier will manifest disease. The potential impact of reduced penetrance on carrier-frequency and ARC estimates was instead assessed post hoc via a sensitivity analysis excluding four well-characterized hypomorphic alleles.

Population frequencies were compared against gnomAD exomes v2.1.1 (hg38, obtained from UCSC Genome Browser). This version was selected because a reduced, locally accessible dataset was available through UCSC Genome Browser, allowing extraction and manipulation of the allele counts required for Fisher’s exact tests. Although newer gnomAD releases, including v4.1.0, are available, an equivalent reduced dataset suitable for our local analysis workflow was not available. The gnomAD genomes v3.0 dataset (also obtained from UCSC Genome Browser as a reduced, locally accessible dataset) was used separately for variant-level filtering based on its FILTER status. Variants with one or more homozygous individuals in the gnomAD v2.1.1 control cohort (*n* = 72, “controls_nhomalt”) and at least one pathogenic ClinVar submission were manually reviewed, and 39 were retained. Variants with a local population allele frequency > 5% were excluded.

For variant quality, genotype quality (GQ) and allele-depth ratio were assessed per GATK output. Variants for which more than 50% of heterozygous calls had GQ < 20, or more than 50% had an allele-depth ratio < 0.35 or >0.75, were flagged as insufficient quality and excluded. Variants failing the gnomAD genomes v3.0 FILTER (i.e., without a PASS annotation) were also removed.

All variants reaching an allele frequency ≥ 1/200 (≈carrier frequency ≥ 1/100) in either of the two largest local ethnic groups (Macedonian and Albanian) underwent additional manual ACMG classification.

### 2.5. Statistical Analysis

Allele frequencies were calculated across all 1438 subjects for genes covered by the clinical exome panel (~4800 genes), and across the 1160 WES subjects for genes captured only by WES. For X-chromosome variants, allele frequencies accounted for subject sex (total allele number = 2 × female subjects + male subjects). Alleles directly responsible for a participant’s primary diagnostic condition (referral reason) were systematically excluded prior to population frequency calculations. For each excluded diagnostic allele, both the allele count (AC) and total evaluated allele number (AN) for that specific variant was adjusted accordingly. Local population allele frequencies (after excluding disease-causing alleles) were compared against gnomAD exomes v2.1.1 non-Finnish European (NFE) frequencies using Fisher’s exact test, with Bonferroni correction for multiple testing.

At-risk-couple (ARC) probability was estimated by summing the allele frequencies of pathogenic variants within each gene to obtain a gene-level allele frequency, converted to carrier frequency via the Hardy–Weinberg relationship (carrier frequency ≈ 2 × allele frequency), which was then squared to estimate the probability of carrier–carrier pairing (ARC) in the population [27]. Genes were ranked in descending order of gene-level ARC, cumulative ARC was computed across the ranked list, and each gene’s fractional contribution to total ARC was calculated (gene-level ARC ÷ total ARC). Because the carrier-pairing model assumes autosomal recessive inheritance, X-linked genes among the qualifying panel genes (6/627 in the combined cohort, 4/534 in the Macedonian cohort, and 1/297 in the Albanian cohort; <1% in each cohort) were excluded from the ARC ranking, cumulative ARC curves, and Supplementary Files S5–S7. Given their small number and the sex-specific nature of X-linked recessive transmission risk, these genes are instead reported separately using allele and carrier frequencies, without at-risk-couple modeling.

Statistical power to detect ≥1 heterozygous carrier of a given gene-level carrier rate (GCR) in each cohort was estimated as Power = 1 − (1 − GCR)^n^, for *n* = 1438 (combined), 972 (Macedonian) and 358 (Albanian) subjects [18] (Supplementary File S3).

All statistical analyses and graphics were produced in R v4.2.1.

## 3. Results

### 3.1. Variant Filtering and Pathogenic/Likely Pathogenic Variant Landscape

Filtering across the 1438 study participants identified 2007 pathogenic or likely pathogenic (P/LP) variants distributed across 1096 genes (Supplementary File S2). While the vast majority of variants were detected across both sequencing strategies, a subset (~16%) was captured exclusively by whole-exome sequencing. Over half of the curated genes (627 genes; 1246 variants) overlapped with our candidate carrier screening panel and were prioritized for downstream ARC modeling, with the remaining genes retained for population-level frequency comparisons.

Across the full curated gene set, we observed 5304 pathogenic alleles. Total pathogenic allele burden was heavily concentrated in a small group of recurrently mutated loci, led by *RBM8A*, *BCHE*, *ABCA4*, *WNT10A*, and *BTD* (Supplementary File S2). Except for *ABCA4*, the overall allele burden in these top-ranked loci was overwhelmingly driven by a single predominant variant with a high relative contribution, most of which are hypomorphic in nature. In contrast, genes such as *ABCA4*, *USH2A*, *PAH*, and *CFTR* displayed the highest allelic heterogeneity, harboring the largest number of distinct P/LP variants across the cohort.

#### Comparison with gnomAD Exomes V2.1.1 (NFE) Allele Frequencies

Comparison of local allele frequencies against gnomAD non-Finnish European (NFE) controls revealed significant population-specific divergence following strict Bonferroni correction for multiple testing. Overall, 6.5% of evaluated variants differed significantly from NFE reference frequencies. Stratification by ethnic background highlighted marked genomic divergence in the Albanian subgroup, where over 10% of identified variants differed significantly from gnomAD NFE, compared to 4.0% in the Macedonian subgroup (Supplementary File S2). This substantially higher proportion of allele-frequency-differentiated variants in the Albanian subgroup—despite fewer total variants evaluated—underscores greater genetic divergence from broader European populations.

### 3.2. Variants Across All Genes

#### Population-Specific High-Frequency Variants by Organ System

**Figure 1:**
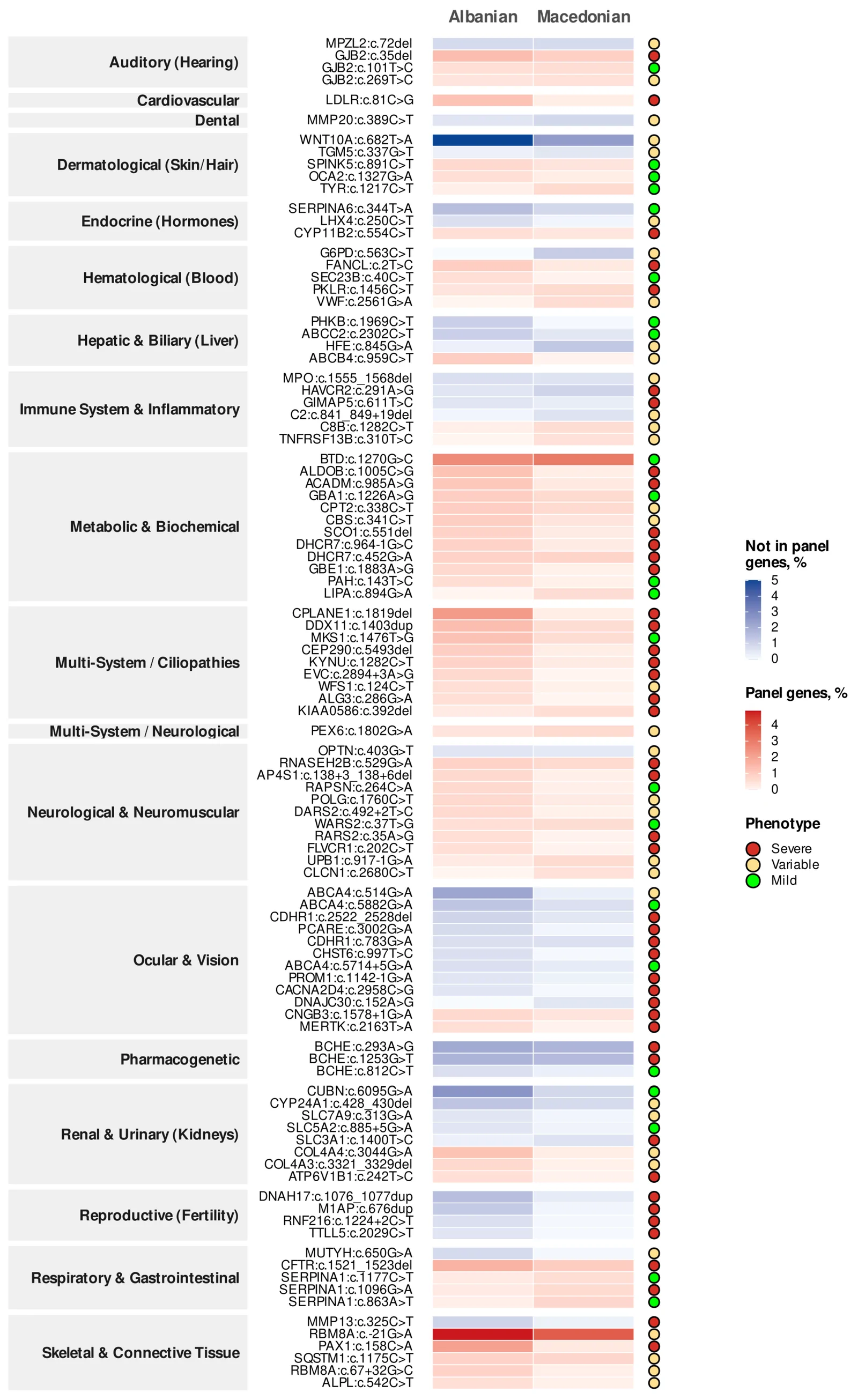
Population-specific allele frequency heatmap of high-frequency P/LP variants (*n* = 100, allele frequency ≥ 1/200 in MK and/or AL), grouped by primary affected organ/system. Tile color intensity indicates magnitude of the allele frequency (%); gene panel-included variants are shown on a red scale, non-panel variants on a blue scale. Circles on the right indicate phenotype severity level within the disease group (red = severe, yellow = variable, and green = mild). Underlying data are provided in Supplementary File S4, allowing readers to reproduce or re-filter the Figure (e.g., excluding Mild-classified conditions).

To compare generic versus population-tailored screening, we selected all ClinVar P/LP variants reaching an allele frequency ≥ 1/200 (carrier frequency ≥ 1/100) in either the Macedonian or Albanian subgroup, yielding 100 high-frequency variants across 89 genes (Figure 1). Grouped by primary affected organ system, the Metabolic and Biochemical, Multi-System/Ciliopathies, and Neurological and Neuromuscular categories contained the highest concentration of qualifying variants in which virtually all variants (with a single exception) belonged to candidate panel genes. Within these panel-heavy categories, *BTD*:c.1270G>C was the single most frequent variant, though it represents a well-characterized hypomorphic allele. Notably, the next most frequent locus in these categories was *CPLANE1*:c.1819del, which reached a high allele frequency specifically in the Albanian subgroup and is associated with a severe ciliopathy phenotype.

Across the entire cohort, the overall highest allele frequencies were driven by *WNT10A*:c.682T>A (dermatological) and *RBM8A*:c.-21G>A (skeletal), both reaching peak frequencies in the Albanian subgroup (~5%). Crucially, neither of these top-ranking variants is associated with life-threatening or life-limiting conditions, carrying a variable rather than severe phenotypic classification (Figure 1). For *RBM8A*:c.-21G>A, homozygosity alone is not a prerequisite for disease manifestation; rather, this hypomorphic allele produces thrombocytopenia-absent radius (TAR) syndrome only when inherited in trans with a structural microdeletion or null mutation [28].

Of the 100 high-frequency variants, 61 were captured by our candidate panel (red-scale tiles, Figure 1), whereas 39 met the carrier-frequency threshold but fell outside it (blue-scale tiles). Importantly, the vast majority of these unrepresented variants (30/39; ~77%) carry a variable or severe phenotype classification, demonstrating that most non-panel high-frequency alleles are clinically significant rather than mild. The Ocular and Vision category contained the largest share of these severe/variable non-panel variants (*n* = 7), establishing it as a primary priority for panel expansion.

As one example among non-panel genes, *MMP13:c.325C>T* (0.98% in AL) is a nonsense variant, p.(Arg109Ter), causing autosomal recessive metaphyseal anadysplasia (MANDP2), a chondrodysplasia presenting with early-onset short stature and metaphyseal/epiphyseal dysplasia. Dominant MMP13 variants are generally associated with a more severe clinical course, whereas recessive variants are classically described as producing a milder phenotype that improves with age; however, the two Albanian brothers in whom this exact variant was independently reported as a homozygous founder mutation showed persistent short stature and mixed epiphyseal and metaphyseal dysplasia into adulthood rather than the expected improvement with age [29]. This locally frequent, clinically significant variant outside the current panel underscores the value of population-specific frequency data over generic screening recommendations.

### 3.3. Variants in Panel Genes

#### 3.3.1. ARC Accumulation, ACMG Concordance, and Subgroup Divergence

**Figure 2:**
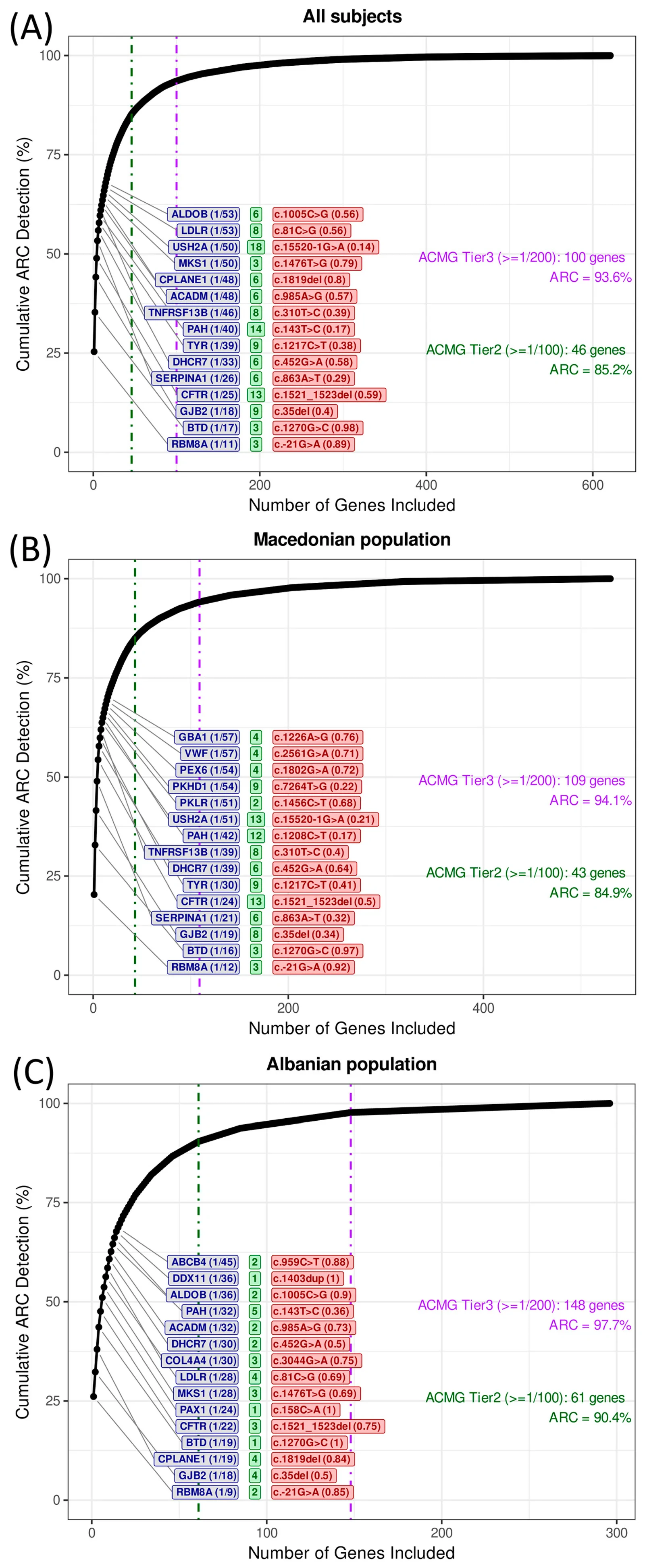
Cumulative at-risk-couple (ARC) detection curves across ranked candidate panel genes for (**A**) the combined cohort (*n* = 1438), (**B**) MK (*n* = 972), and (**C**) AL (*n* = 358). Genes with ≥1 qualifying P/LP variant were ranked by individual ARC contribution and plotted against cumulative % of total ARC detected (black curve), yielding a saturating, Pareto-type distribution in each (621, 530, and 296 qualifying autosomal recessive genes, panels (**A**–**C**), respectively; Supplementary Files S5–S7). X-linked genes identified among the qualifying panel genes were excluded from these curves and are reported separately in Supplementary Files S5–S7. Green and purple dashed lines mark ACMG Tier 2 (≥1/100) and Tier 3 (≥1/200) thresholds, with gene counts and cumulative ARC given alongside. For the top 15 genes per cohort, we have the following: blue labels give gene symbol and carrier frequency (“1 in N”); green labels give the number of distinct qualifying P/LP variants; red labels give the single most frequent variant and its relative contribution to gene-level carrier frequency. Only 6 of the top 15 genes were shared between panels (**B**,**C**).

Ranking genes by individual at-risk-couple (ARC) contribution yielded saturating, Pareto-type cumulative detection curves across all cohorts (Figure 2A–C, Supplementary Files S5–S7). In both the combined cohort and ethnic subgroups, the top 15 ranked genes alone accounted for roughly two-thirds of total cumulative ARC. Applying ACMG carrier-frequency tiering, Tier 2 (≥1/100) and Tier 3 (≥1/200) thresholds captured the vast majority of cumulative ARC in all three cohorts (~85–90% and ~94–98%, respectively).

However, population-specific top-ranking genes exhibited substantial discordance with standard pan-ethnic screening panels. Notably, roughly half of the top 15 ARC-contributing genes in our population—including loci associated with severe, prenatal-lethal, or highly disabling conditions such as *CPLANE1*, *DDX11*, *PAX1*, *PKLR*, *PEX6*, and *GBA1*—are completely absent from the 113-gene ACMG pan-ethnic list. At the full candidate-gene level, only 75/627 (combined), 72/534 (MK), and 49/297 (AL) genes overlapped with ACMG-113 at all. Furthermore, subgroup comparisons revealed marked ethnicity-driven ranking divergence: only 6 of the top 15 ARC-contributing genes were shared between Macedonians and Albanians (Supplementary Files S6 and S7), reinforcing the limitations of universal, non-stratified panel designs.

#### 3.3.2. Allelic Architecture, Disease Incidence, and Sensitivity Analysis of Hypomorphic Variants

Analysis of allelic architecture among top-ranking loci revealed two distinct patterns (Figure 2A–C). Several high-ARC genes showed carrier frequencies driven almost entirely by a single recurrent founder variant (e.g., *BTD*, *RBM8A*, *CPLANE1*, *PAX1*, and *DDX11*), making them amenable to low-cost targeted genotyping. Conversely, loci such as *USH2A*, *PAH*, and *PKHD1* exhibited broad allelic spectra requiring full-exon sequencing.

Across all qualifying autosomal recessive panel genes (X-linked genes excluded; see Methods), cumulative baseline ARC was 3.42% (1 in 29 couples) in the combined cohort, corresponding to an estimated theoretical maximum recessive-disease incidence of 0.86% (1 in 117 pregnancies) under full-penetrance assumptions. Cumulative ARC was lower in Macedonians (3.16%; 1 in 32) and markedly higher in Albanians (5.02%; 1 in 20) (Supplementary Files S5–S7). These are estimated probabilities under Hardy–Weinberg equilibrium and random-mating assumptions, not directly measured risks.

Because several leading variants represent well-known hypomorphic alleles (*RBM8A*:c.-21G>A, *BTD*:c.1270G>C, *MKS1*:c.1476T>G, and *PEX6*:c.1802G>A), we performed a sensitivity analysis excluding these four specific variants (Table 4). This exclusion resulted in a relative reduction of 33.7–36.8% in cumulative ARC across all cohorts, lowering adjusted ARC to 2.16% (1 in 46) in the combined cohort, 2.07% (1 in 48) in Macedonians, and 3.33% (1 in 30) in Albanians. Crucially, the fold-difference between the Albanian and Macedonian subgroups remained essentially identical before (1.59-fold) and after (1.60-fold) exclusion. This demonstrates that while hypomorphic alleles contribute substantially to baseline screening yield, they do not account for the specifically elevated ARC observed in the Albanian subgroup, which is instead driven by non-hypomorphic, population-enriched loci such as *CPLANE1*, *PAX1*, *COL4A4*, *DDX11*, and *ABCB4*.

Statistical power to detect at least one carrier was ≥99% at the ACMG Tier 3 threshold (GCR ≥ 1/200) in the combined and Macedonian cohorts, but lower in the smaller Albanian subgroup (83%); power fell below 80% for GCR ≤ 1/500 in the Albanian subgroup, and for GCR ≤ 1/1000 in the combined and Macedonian cohorts (Supplementary File S3).

## 4. Discussion

This study characterized the pathogenic/likely pathogenic (P/LP) variant landscape across a large, ethnically stratified cohort from North Macedonia and used it to model at-risk-couple (ARC) probability for expanded carrier screening (ECS) panel design. Consistent with prior ECS cohorts, ARC contribution across genes followed a saturating, Pareto-type distribution, with a small subset of high-frequency genes accounting for the majority of cumulative risk [27,30]. However, the substantial discordance between our top ARC-contributing genes and the ACMG Tier 3 pan-ethnic list—together with the marked divergence in gene ranking between the Macedonian and Albanian subgroups—indicates that a generic, gnomAD-derived panel would systematically under-detect ARC in this population. These findings reinforce a central premise of population-tailored ECS design: gene selection informed by local allele-frequency data captures a materially different, and more locally relevant, set of at-risk couples than pan-ethnic reference panels alone.

Our approach to panel design aimed to balance systematic coverage of well-characterized, clinically actionable recessive disorders with a broader assessment of genetic risk to reproductive outcomes. Beyond the postnatal, phenotype-defined conditions that dominate conventional ECS gene lists, we deliberately incorporated genes implicated in embryonic and fetal lethality. Variants in these genes are a recognized, though under-ascertained, contributor to recurrent pregnancy loss and early fetal demise, and their omission from standard carrier panels likely underestimates the true reproductive genetic burden in any population, including ours. Their inclusion here is intended to complement, not replace, phenotype-focused panel design, providing a more complete picture of recessive reproductive risk.

Phenotype severity does not align neatly with panel status. For instance, as described earlier, *MMP13*:c.325C>T exhibits high local frequency and pronounced clinical impact despite its omission from standard panels, whereas candidate panels often include pathogenic variants with much milder presentations. Furthermore, ocular and vision phenotypes emerged as the most systematically underrepresented category among non-panel, high-frequency variants in our dataset—a gap that may warrant dedicated attention in future panel iterations.

Two neighboring Balkan/Central European studies offer external context. *GJB2*, *CFTR*, *DHCR7*, and *PAH* were among the highest carrier-frequency genes in a large Slovenian genomic cohort, overlapping with our own top-ranked genes, while *CNGA1*—third-highest in Slovenia—was notably absent from our high-ARC genes, reflecting a shared regional core alongside genuine population-specific divergence [11]. In a Greek cohort screened against the ACMG/ACOG 176-gene panel, the overall ARC rate was 1.6% (1 in 60 couples) [8], roughly half our combined-cohort estimate (3.42%, 1 in 29 couples)—plausibly reflecting our broader, non-ACMG-restricted candidate gene set.

Where comparable population-specific data exist, our carrier-frequency estimates align well with earlier, independently generated data from our region. For cystic fibrosis, the *CFTR* carrier frequency observed here is consistent with earlier molecular screening of the Macedonian population, including a targeted mutation-panel study in infertile men [31] and a national CF registry-based mutation spectrum analysis [32], both of which used minisequencing or targeted genotyping rather than exome-wide sequencing. This concordance across independent methodologies and cohorts supports the reliability of our exome-derived carrier-frequency estimates for well-studied genes.

Among the top-ranked genes by carrier frequency, we identified—alongside well-recognized recessive Mendelian disorders such as cystic fibrosis and phenylketonuria—several genes with comparably high predicted carrier frequencies and expected disease incidences for which no or a small number of affected patients have been observed in our internal diagnostic records. This discrepancy between predicted and observed disease burden is unlikely to reflect incomplete ascertainment alone, and instead points to two non-mutually exclusive explanations.

First, some of the underlying variants may be associated with prenatal or early embryonic lethality, such that affected conceptions are lost before clinical presentation and therefore never enter a postnatal diagnostic pathway; candidate examples in our dataset include *CPLANE1:c.1819del*, *DHCR7:c.1403dup*, and *DDX11:c.452G>A*. Direct support for this comes from our concurrent whole-exome sequencing studies of euploid products of conception from early pregnancy loss in the same population [33,34], in which biallelic *CPLANE1* and *DHCR7* variants were the leading molecular diagnoses established in the conceptus. Notably, the *CPLANE1* finding in that cohort was a complex allele combining *c.1819delT* in cis with *c.7817T>A*, recurrent across unrelated families—particularly of Albanian ancestry—and corresponding directly to the *CPLANE1:c.1819del* variant among our own top ARC-contributing alleles here; *DHCR7* compound heterozygosity for severe, Smith–Lemli–Opitz-associated alleles was likewise recurrent among these fetal losses [33]. These concordant, population-matched findings support prenatal or early embryonic lethality as a plausible explanation for the apparent absence of postnatal cases in genes such as *CPLANE1* and *DHCR7*.

Second, some variants may instead be hypomorphic, producing milder or atypical phenotypes that go unrecognized or are misattributed to other causes—a discrepancy between predicted carrier frequency and clinically ascertained disease burden that recurs among several of our own leading variants. *BTD:c.1270G>C*, *p.(Asp444His)*—our leading gene-level variant by pathogenic allele count, accounting for 0.98 of the carrier frequency of BTD, and also discussed in the Greek cohort referenced above—is itself a well-recognized hypomorphic allele, producing partial biotinidase deficiency that is often mild and may escape ascertainment outside newborn screening. *RBM8A:c.-21G>A*, the single most frequent leading variant in both our MK (relative contribution 0.92) and AL (0.85) subgroups, is an established hypomorphic 5′UTR allele that only causes thrombocytopenia-absent radius (TAR) syndrome when inherited in trans with a null *RBM8A* allele or the recurrent 1q21.1 microdeletion [28]; heterozygosity for this allele alone is not disease-causing, which explains its markedly high carrier frequency without a corresponding excess of TAR syndrome cases in our population. *MKS1:c.1476T>G*, the leading *MKS1* variant in both subgroups, has similarly been detected in unaffected homozygotes and shown to mildly impair ciliogenesis in vitro [35], supporting its classification as a hypomorphic allele and accounting for why its predicted carrier frequency is not matched by a corresponding number of clinically ascertained Meckel/Joubert-spectrum cases in our data. A directly analogous example was reported in a Greek ECS cohort, where an unexpectedly high *PEX6* carrier frequency was attributed to a hypomorphic variant associated with a mild, likely under-diagnosed late-onset phenotype [8]. *PEX6:c.1802G>A*, the leading *PEX6* variant among our own MK top-15 genes, corresponds to the well-documented hypomorphic *PEX6 p.(Arg601Gln)* allele associated with mild, often late-onset Zellweger spectrum and Heimler syndrome phenotypes [36], which indicates that this is the recurrent allele driving elevated PEX6 carrier frequency across the region. Importantly, our sensitivity analysis demonstrated that removing these four recurrent hypomorphic variants results in a substantial reduction in cumulative ARC across all cohorts. Crucially, however, this exclusion left the relative ARC excess in the Albanian subgroup compared to Macedonians virtually unchanged, confirming that while hypomorphic alleles substantially inflate baseline screening yields overall, they do not account for the population-specific risk elevation observed in Albanians. Distinguishing hypomorphic alleles from classical severe mutations is therefore essential for accurate clinical risk communication and panel prioritization.

Several limitations should be considered. Our cohort, while large, remains underpowered for ultra-rare or private pathogenic variants, so calculated ARC frequencies are likely conservative. Formal power calculations confirm this: power to detect ≥1 carrier exceeded 99% at the ACMG Tier 3 threshold in the combined and Macedonian cohorts but was more limited in the smaller Albanian subgroup and for rarer variants generally. Reliance on whole-exome sequencing limits detection of structural variants, large copy-number changes, and deep intronic variants relevant to recessive disease and pregnancy loss. Variant calling was further restricted to coding exons and a 25 bp flanking region on each side of targeted exons; any pathogenic variant located outside this window—including deep intronic and other non-captured regulatory variants—would therefore have been missed. Our ARC and incidence values are estimated probabilities that assume Hardy–Weinberg equilibrium and panmixia, which may underestimate true frequencies in subpopulations with meaningful substructure or consanguinity. The penetrance and expressivity of embryonic/fetal-lethality genes, curated from the literature, can vary substantially and warrant cautious individual interpretation. Our cohort was also ascertained through diagnostic referral rather than unselected population sampling, which—despite excluding each subject’s primary diagnostic alleles—could still bias the represented variant spectrum. This bias likely acts in both directions: genes overlapping common referral indications may be over-represented, while genes unrelated to those indications may be under-ascertained, relative to a true population sample. Our estimates should therefore be understood as cohort-derived, consistent with how such data are used in the literature, and confirmation in a broader population-based sample would further strengthen their application to individual-level reproductive risk counseling. Additionally, standard ARC modeling treats all variants as fully penetrant, whereas a small number of hypomorphic alleles accounted for a major fraction of our cumulative risk. Failing to adjust for hypomorphic mechanisms or reduced penetrance risks overestimating true actionable reproductive risk, underscoring the need for penetrance-weighted screening models. Because the at-risk-couple framework applied here models autosomal recessive carrier–carrier pairing, the small number of X-linked genes identified among the qualifying panel genes (<1% of qualifying genes, variants, and carrier observations in each cohort) could not be incorporated into the same ARC and incidence calculations and were instead reported separately as allele and carrier frequencies; a dedicated, sex-stratified X-linked risk model was beyond the scope of the present analysis. Finally, gnomAD exomes v2.1.1 NFE is not a fully ancestry-matched comparator for Balkan populations, and, together with the broader underrepresentation of non-Northern-European populations in ClinVar [37], this raises the possibility that some locally relevant pathogenic variants remain unrecognized in either database and are consequently absent from our curated variant list.

Taken together, these findings support a population-informed approach to expanded carrier screening panel design in North Macedonia—one that combines standardized, pan-ethnic frameworks such as ACMG Tier 3 with locally derived carrier-frequency data. Given their demonstrated contribution to estimated reproductive-risk probability in this population, we consider it advisable to incorporate genes associated with embryonic and fetal lethality into ECS panel design, complementing rather than replacing conventional, phenotype-focused gene lists. Panel design and risk communication should equally account for the presence of hypomorphic alleles, whose reduced penetrance can substantially inflate predicted carrier frequencies without a corresponding excess of clinically ascertained disease, so that actionable reproductive risk is not overestimated. Such a population-tailored, penetrance-aware approach stands to improve ARC detection sensitivity for this specific population while also generating longitudinal carrier-frequency data of value to broader public health and reproductive care planning.

## Figures and Tables

**Table 1:** Initial cohort by enrichment strategy, exclusions, and final cohort composition.

Enrichment Strategy	Platform	Total (Initial)	Excluded	Final (*n*)	Final (%)
Clinical exome	Illumina TruSight One	291	13	278	19.3%
WES	Twist Bioscience (Human Core + RefSeq + Mitochondrial)	1164	79	1085	75.5%
WES	Illumina Exome Panel v1.2	77	2	75	5.2%
Total		1532	94	1438	100%

Of the final cohort, 48% (*n* = 690) were female and 52% (*n* = 748) were male.

**Table 2:** Ethnic distribution of the final cohort (*n* = 1438).

Ethnicity	*n*	%
Macedonian	972	67.5%
Albanian	358	25.0%
Roma	39	2.7%
Turkish	26	1.8%
Other	43	3.0%
Total	1438	100%

**Table 3:** Sources contributing to the candidate gene list.

Source	Number of AR/XLR Genes	Reference
Literature		
ACMG pan-ethnic carrier screening practice resource	113	(Gregg et al., 2021) [6]
Miscarriage risk assessment: candidate lethal genes and variants	51	(Aminbeidokhti et al., 2024) [16]
Lethal phenotypes in Mendelian disorders	289	(Cacheiro et al., 2024) [17]
Gene discovery toolkit for unexplained infertility and prenatal/infantile mortality	244	(Dawes et al., 2019) [13]
PanelApp		
Mackenzie’s Mission—Reproductive Carrier Screening (v0.111, PanelApp Australia, green list)	1283	(Kirk et al., 2021) [15]
Fetal Anomalies (v1.470, PanelApp Australia, green list)	921	(Lord et al., 2019) [14]
Fetal Anomalies (v6.116, PanelApp UK, green list)	960	(Lord et al., 2019) [14]
Total unique genes after merging	1795	

**Table 4:** Sensitivity analysis of cumulative at-risk-couple (ARC) probability following exclusion of leading hypomorphic variants.

Cohort	Baseline ARC, % (1 in N)	Adjusted ARC *, % (1 in N)	Absolute Reduction (pp)	Relative Reduction (%)
Combined	3.42% (1 in 29)	2.16% (1 in 46)	1.26 pp	36.84%
Macedonian (MK)	3.16% (1 in 32)	2.07% (1 in 48)	1.09 pp	34.49%
Albanian (AL)	5.02% (1 in 20)	3.33% (1 in 30)	1.69 pp	33.67%

* Adjusted ARC calculated after excluding four high-frequency hypomorphic variants. pp = percentage points.

## Data Availability

Further inquiries can be directed to the corresponding author.

## Supplementary Materials

The following supporting information can be downloaded at: https://www.mdpi.com/article/10.3390/genes17091118/s1; Supplementary File S1: List of the 1795 candidate autosomal recessive/X-linked genes and their contributing sources; Supplementary File S2: Full list of curated pathogenic/likely pathogenic (P/LP) variants (*n* = 2007) with population allele frequencies and gnomAD comparison statistics; Supplementary File S3: Short methodology and results for calculated statistical power, by cohort, to detect at least one heterozygous carrier at given population gene carrier rates; Supplementary File S4: Underlying data for Figure 1 (*n* = 100 high-frequency P/LP variants, allele frequencies, primary organ/system, panel status, and phenotype severity classification), provided so that the Figure can be reproduced or re-filtered (e.g., excluding Mild-classified conditions); Supplementary Files S5–S7: Gene-level at-risk-couple (ARC) ranking and cumulative ARC data underlying Figure 2 for the combined cohort (S5), Macedonian subgroup (S6), and Albanian subgroup (S7), each including a separate sheet listing allele and carrier frequencies for the X-linked genes excluded from the ARC model.

## References

[1] American College of Obstetricians and Gynecologists, Committee on Genetics (2017). Committee Opinion No. 691: Carrier Screening for Genetic Conditions. Obstet. Gynecol..

[2] Henneman L., Borry P., Chokoshvili D., Cornel M.C., van El C.G., Forzano F., Hall A., Howard H.C., Janssens S., Kayserili H. (2016). Responsible implementation of expanded carrier screening. Eur. J. Hum. Genet..

[3] Edwards J.G., Feldman G., Goldberg J., Gregg A.R., Norton M.E., Rose N.C., Schneider A., Stoll K., Wapner R., Watson M.S. (2015). Expanded carrier screening in reproductive medicine-points to consider: A joint statement of the American College of Medical Genetics and Genomics, American College of Obstetricians and Gynecologists, National Society of Genetic Counselors, Perinatal Quality Foundation, and Society for Maternal-Fetal Medicine. Obstet. Gynecol..

[4] Van Steijvoort E., Chokoshvili D., Cannon J.W., Peeters H., Peeraer K., Matthijs G., Borry P. (2020). Interest in expanded carrier screening among individuals and couples in the general population: Systematic review of the literature. Hum. Reprod. Update.

[5] Pilenzi L., Scorrano V., Di Rado S., Buccolini C., Giansante R., Siciliani L., Stuppia L., Gatta V., Capalbo A. (2026). Expanded Carrier Screening: Current Evidence and Future Directions in the Era of Population Genomics. Genes.

[6] Gregg A.R., Aarabi M., Klugman S., Leach N.T., Bashford M.T., Goldwaser T., Chen E., Sparks T.N., Reddi H.V., Rajkovic A. (2021). Screening for autosomal recessive and X-linked conditions during pregnancy and preconception: A practice resource of the American College of Medical Genetics and Genomics (ACMG). Genet. Med..

[7] Schmitz M.J., Bashar A., Soman V., Nkrumah E.A.F., Al Mulla H., Darabi H., Wang J., Kiehl P., Sethi R., Dungan J. (2025). Leveraging diverse genomic data to guide equitable carrier screening: Insights from gnomAD v.4.1.0. Am. J. Hum. Genet..

[8] Marinakis N.M., Tilemis F.N., Veltra D., Svingou M., Sofocleous C., Kekou K., Kosma K., Kampouraki A., Kontse C., Fylaktou I. (2025). Estimating at-risk couple rates across 1000 exome sequencing data cohort for 176 genes and its importance relevance for health policies. Eur. J. Hum. Genet..

[9] Tan J., Jiang Z., Shao B., Wang Y., Zhang J., Hu P., Luo C., Xu Z. (2025). Expanded carrier screening for 224 monogenic disease genes in 1,499 Chinese couples: A single-center study. Clin. Chem. Lab Med..

[10] Kovacevic L., Tambets K., Ilumäe A.M., Kushniarevich A., Yunusbayev B., Solnik A., Bego T., Primorac D., Skaro V., Leskovac A. (2014). Standing at the gateway to Europe—The genetic structure of Western balkan populations based on autosomal and haploid markers. PLoS ONE.

[11] Maver A., Juvan P., Kotnik U., Lovrecic L., Bergant G., Peterlin B. (2025). Creating the Slovenian genome database and browser as a source of comprehensive variation of the Slovenian population. Sci. Rep..

[12] Gambin T., Jhangiani S.N., Below J.E., Campbell I.M., Wiszniewski W., Muzny D.M., Staples J., Morrison A.C., Bainbridge M.N., Penney S. (2015). Secondary findings and carrier test frequencies in a large multiethnic sample. Genome Med..

[13] Dawes R., Lek M., Cooper S.T. (2019). Gene discovery informatics toolkit defines candidate genes for unexplained infertility and prenatal or infantile mortality. npj Genom. Med..

[14] Lord J., McMullan D.J., Eberhardt R.Y., Rinck G., Hamilton S.J., Quinlan-Jones E., Prigmore E., Keelagher R., Best S.K., Carey G.K. (2019). Prenatal exome sequencing analysis in fetal structural anomalies detected by ultrasonography (PAGE): A cohort study. Lancet.

[15] Kirk E.P., Ong R., Boggs K., Hardy T., Righetti S., Kamien B., Roscioli T., Amor D.J., Bakshi M., Chung C.W.T. (2021). Gene selection for the Australian Reproductive Genetic Carrier Screening Project (“Mackenzie’s Mission”). Eur. J. Hum. Genet..

[16] Aminbeidokhti M., Qu J.H., Belur S., Cakmak H., Jaswa E., Lathi R.B., Sirota M., Snyder M.P., Yatsenko S.A., Rajkovic A. (2024). Miscarriage risk assessment: A bioinformatic approach to identifying candidate lethal genes and variants. Hum. Genet..

[17] Cacheiro P., Lawson S., Van den Veyver I.B., Marengo G., Zocche D., Murray S.A., Duyzend M., Robinson P.N., Smedley D. (2024). Lethal phenotypes in Mendelian disorders. Genet. Med..

[18] Gruzin M.J., Hobbs M., Ellsworth R.E., Poll S., Aguilar S., Knezovich J., Faulkner N., Olsen N., Aradhya S., Burnett L. (2025). Optimizing gene panels for equitable reproductive carrier screening: The Goldilocks approach. Genet. Med..

[19] Shraga R., Yarnall S., Elango S., Manoharan A., Rodriguez S.A., Bristow S.L., Kumar N., Niknazar M., Hoffman D., Ghadir S. (2017). Evaluating genetic ancestry and self-reported ethnicity in the context of carrier screening. BMC Genet..

[20] Feero W.G., Steiner R.D., Slavotinek A., Faial T., Bamshad M.J., Austin J., Korf B.R., Flanagin A., Bibbins-Domingo K. (2024). Guidance on use of race, ethnicity, and geographic origin as proxies for genetic ancestry groups in biomedical publications. Hum. Genet. Genom. Adv..

[21] Kaseniit K.E., Haque I.S., Goldberg J.D., Shulman L.P., Muzzey D. (2020). Genetic ancestry analysis on >93,000 individuals undergoing expanded carrier screening reveals limitations of ethnicity-based medical guidelines. Genet. Med..

[22] Li H. (2013). Aligning sequence reads, clone sequences and assembly contigs with BWA-MEM. arXiv.

[23] McKenna A., Hanna M., Banks E., Sivachenko A., Cibulskis K., Kernytsky A., Garimella K., Altshuler D., Gabriel S., Daly M. (2010). The Genome Analysis Toolkit: A MapReduce framework for analyzing next-generation DNA sequencing data. Genome Res..

[24] McLaren W., Gil L., Hunt S.E., Riat H.S., Ritchie G.R., Thormann A., Flicek P., Cunningham F. (2016). The Ensembl Variant Effect Predictor. Genome Biol..

[25] Stark Z., Foulger R.E., Williams E., Thompson B.A., Patel C., Lunke S., Snow C., Leong I.U.S., Puzriakova A., Daugherty L.C. (2021). Scaling national and international improvement in virtual gene panel curation via a collaborative approach to discordance resolution. Am. J. Hum. Genet..

[26] Stawiński P., Płoski R. (2024). Genebe.net: Implementation and validation of an automatic ACMG variant pathogenicity criteria assignment. Clin. Genet..

[27] Guo M.H., Gregg A.R. (2019). Estimating yields of prenatal carrier screening and implications for design of expanded carrier screening panels. Genet. Med..

[28] Albers C.A., Paul D.S., Schulze H., Freson K., Stephens J.C., Smethurst P.A., Jolley J.D., Cvejic A., Kostadima M., Bertone P. (2012). Compound inheritance of a low-frequency regulatory SNP and a rare null mutation in exon-junction complex subunit RBM8A causes TAR syndrome. Nat. Genet..

[29] Li D., Weber D.R., Deardorff M.A., Hakonarson H., Levine M.A. (2015). Exome sequencing reveals a nonsense mutation in MMP13 as a new cause of autosomal recessive metaphyseal anadysplasia. Eur. J. Hum. Genet..

[30] Ben-Shachar R., Svenson A., Goldberg J.D., Muzzey D. (2019). A data-driven evaluation of the size and content of expanded carrier screening panels. Genet. Med..

[31] Noveski P., Madjunkova S., Mircevska M., Plaseski T., Filipovski V., Plaseska-Karanfilska D. (2014). SNaPshot assay for the detection of the most common CFTR mutations in infertile men. PLoS ONE.

[32] Terzic M., Jakimovska M., Fustik S., Jakovska T., Sukarova-Stefanovska E., Plaseska-Karanfilska D. (2019). Cystic Fibrosis Mutation Spectrum in North Macedonia: A Step Toward Personalized Therapy. Balk. J. Med. Genet..

[33] Bozhinovski G., Noveski P., Terzikj M., Kubelka-Sabit K., Plaseska-Karanfilska D. (2025). Monogenic Findings in Early Pregnancy Loss: Whole-Exome Sequencing Study of Euploid Products of Conception. Balk. J. Med. Genet..

[34] Bozhinovski G., Terzikj M., Kubelka-Sabit K., Plaseska-Karanfilska D. (2024). High Incidence of CPLANE1-Related Joubert Syndrome in the Products of Conceptions from Early Pregnancy Losses. Balk. Med. J..

[35] Serpieri V., Mortarini G., Loucks H., Biagini T., Micalizzi A., Palmieri I., Dempsey J.C., D’Abrusco F., Mazzotta C., Battini R. (2023). Recurrent, founder and hypomorphic variants contribute to the genetic landscape of Joubert syndrome. J. Med. Genet..

[36] Ratbi I., Falkenberg K.D., Sommen M., Al-Sheqaih N., Guaoua S., Vandeweyer G., Urquhart J.E., Chandler K.E., Williams S.G., Roberts N.A. (2015). Heimler Syndrome Is Caused by Hypomorphic Mutations in the Peroxisome-Biogenesis Genes PEX1 and PEX6. Am. J. Hum. Genet..

[37] Popejoy A.B., Ritter D.I., Crooks K., Currey E., Fullerton S.M., Hindorff L.A., Koenig B., Ramos E.M., Sorokin E.P., Wand H. (2018). The clinical imperative for inclusivity: Race, ethnicity, and ancestry (REA) in genomics. Hum. Mutat..

